# *Bifidobacterium adolescentis* protects against necrotizing enterocolitis and upregulates TOLLIP and SIGIRR in premature neonatal rats

**DOI:** 10.1186/s12887-016-0759-7

**Published:** 2017-01-05

**Authors:** Wenshen Wu, Yanli Wang, Jingjing Zou, Fang Long, Huiheng Yan, Lijuan Zeng, Yunbin Chen

**Affiliations:** 1 Department of Neonatal Intensive Care Unit, Guangdong Province Maternal and Children’s Hospital, Guangzhou Medical University, 13, Guangyuanxi Road, Guangzhou, China; 2 Department of Pediatrics, Guangdong Province Maternal and Children’s Hospital, Guangzhou Medical University, 13, Guangyuanxi Road, Guangzhou, China

**Keywords:** Necrotizing enterocolitis, Toll-like receptor 4, Toll interacting protein, single Ig IL-1-Related receptor

## Abstract

**Background:**

Necrotizing enterocolitis (NEC) is a serious gastrointestinal disorder that is often seen in premature infants. Probiotics decrease the risk of NEC; however, the mechanism by which probiotics work is not clear. The goal of this study was to evaluate the preventive effect of *Bifidobacterium adolescentis* in an NEC rat model.

**Methods:**

Sprague-Dawley neonatal rats were obtained by caesarean section after 20-21 d gestation and randomly divided into the following 3 groups: dam fed (DF), formula fed (FF), and formula + *B. adolescentis* (FB). Those in the FF and FB groups developed NEC after exposure to asphyxia and cold stress. All rats were sacrificed 72 h after birth and intestinal injury and mRNA expression of TLR4, TOLLIP and SIGIRR were assessed.

**Results:**

*B. adolescentis* significantly increased the 72-h survival rate from 56.3% in the FF group to 86.7% in the FB group. *B. adolescentis* significantly reduced the histological score from a median of 3.0 in the FF group to a median of 1.0 in the FB group,and significantly decreased the rate of NEC-like intestinal injury from 77.8% in the FF group to 23.1% in the FB group. The mRNA expression of TLR4 increased 3.6 fold in the FF group but decreased by 2 fold from *B. adolescentis* treatment. mRNA expression of TOLLIP and SIGIRR decreased 4.3 and 3.7 fold, respectively, in the FF group. *B. adolescentis* significantly increased mRNA expression of TOLLIP and SIGIRR by 3.7 fold and 2.6 fold, respectively.

**Conclusions:**

This study demonstrated *B. adolescentis* prevents NEC in preterm neonatal rats and that the mechanism for this action might be associated with the alteration of TLR4, TOLLIP, and SIGIRR expression.

## Background

Necrotizing enterocolitis (NEC) is a serious gastrointestinal disorder that frequently affects premature newborns and results in high morbidity and mortality, especially in very low-birth weight (VLBW) infants [1, 2]. NEC affects approximately 7.0% of all VLBW infants [1] and the death rate associated with the disease is >20%, [1, 3] and nearly one-half of NEC infants need surgery [4]. Premature newborns often must receive long-term parenteral nutrition. These infants often develop NEC and are subsequently at increased risk for short bowel syndrome and cholestatic liver disease [4]. In addition, the premature infants with NEC who need surgery are at increased risk for neurodevelopmental disorders, such as microcephaly and cerebral palsy [5]; therefore, it is important to identify a preventive strategy that will reduce the risk of developing NEC.

Although much research has found that NEC might be a multifactor disease with genetic susceptibility, prematurity, formula feeding and abnormal microbial colonization as risk factors, the exact pathophysiology of the disease remains unknown [1, 6]. NEC pathogenesis is characterized by intestinal inflammation [7] and it was recently suggested that the disease is actually caused by excessive intestinal inflammation, which is associated with an immature innate immune response, such as that in premature infants [8]. Toll-like receptors (TLRs) are a large family of molecules that play a key role in the innate immune response [9]. The ligand of one such TLR, TLR4, is a lipopolysaccharide (LPS), a component of the cell wall of Gram-negative bacteria [9]. TLR4 plays a role in the intestinal immune response; it maintains low levels in mature intestinal epithelium [10] but is overexpressed in human fetal enterocytes [11], which predisposes immature intestinal epithelium to excessive intestinal inflammation when exposed to LPS. The results of a recent study that used an experimental rat model in which NEC was induced by a combination of formula feeding and hypoxia demonstrated that TLR4 mutant mice were more protected from NEC development compared to wild-type mice [10]. It was also reported that intestinal epithelial TLR4 inhibits enterocyte proliferation and migration [12], and that endothelial TLR4 impairs intestinal microcirculatory perfusion [13], suggesting that TLR4 plays an important role in NEC pathogenesis.

TOLLIP and SIGIRR proteins are negative regulators of TLR4 signaling and play an inhibitory role in the TLR-mediated pathway [14, 15]. It was recently reported that they also play an important role in intestinal inflammation [16, 17]. These negative regulators inhibit the signal from TLR4 to downstream pathways by suppressing the activity of interleukin (IL)-1 receptor-associated kinase (IRAK), which is a crucial molecule in the TLR4 signaling pathway [14]. After exposure to dextran sulfate sodium (DSS) for 5.0 d, TOLLIP-deficient mice showed increased severity of intestinal symptoms and greater expression of proinflammatory cytokines compared with wild-type mice [16]. In addition, the level of SIGIRR decreased in ulcerative colitis patients and in the experimental colitis mouse model [17]. There is less TOLLIP and SIGIRR mRNA in immature enterocytes and even less in NEC enterocytes compared with that in mature enterocytes [4]. These results suggest that both TLR4 and its negative regulators may play a critical role in the development of NEC.

*Bifidobacterium* is one type of probiotic, which are live, beneficial microorganisms that predominate in the intestinal flora of breast-fed infants [18]. Recent clinical studies have shown that oral administration of *B. infantis* and *B. bifidum* could prevent NEC development in premature infants [19, 20]. It is not entirely clear how *Bifidobacterium* species protect the neonatal intestine against NEC, but recent reports show that *B. infantis* exerts an anti-inflammatory effect by decreasing TLR4 mRNA and increasing TOLLIP and SIGIRR mRNA levels in immature human intestinal xenografts and NEC intestinal epithelial cells [4]. It is unknown whether *B. adolescentis* has the same effect in the NEC rat model; therefore, the aim of this study was to determine whether *B. adolescentis* could protect against NEC and whether TLR4, TOLLIP and SIGIRR expression could be influenced by *B. adolescentis*.

## Methods

### Animal model

All animal experiments were reviewed and approved by the Animal Care and Welfare Committee of Guangzhou Medical University (Guangzhou, China). All animal experiments were performed in accordance with the U.S. National Institutes of Health’s “Guide for the Care and Use of Laboratory Animals” (NIH Publications No. 80-23 revised 1996). Every effort was made to minimize the number of animals used and reduce suffering. Adult Sprague-Dawley (SD) rats were purchased from Guangdong Medical Laboratory Animal Center (Foshan, China). They were housed at the Guangzhou Medical University Laboratory Animal Center and kept in a room at 22 °C, with a 12-h light-dark cycle [21]. Premature pups were delivered from time-dated pregnant SD rats by Cesarean section 20-21 d of gestation and were reared in incubators at 37 °C [10].

Forty five premature pups were randomly divided into the following three groups: dam fed (DF, *n* = 14), formula fed (FF, *n* = 16), and formula + *B. adolescentis* (FB, *n* = 15). Pups in the DF group were fed by surrogate mothers, and the newborn rats in the FF and FB groups were fed 2.0 h after birth with Esbilac puppy formula (PetAg, Inc., Hampshire, IL, USA). Feeding started with 0.15 mL every 4.0 h and advanced, as tolerated, to a maximum of 0.20 mL per feeding after 24 h of life through a home-made orogastric feeding catheter. Premature newborn rats from the FB group were treated with 1.0 × 10^8^ CFU live *B. adolescentis* per day, (Livzon, Zhuhai, China) diluted in formula.

NEC protocol was followed in the FF and FB groups after the first feeding. Experimental NEC was induced by 90 s of 100% nitrogen exposure and subsequent 10 min cold exposure (4.0 °C) twice daily for 72 h. Rats in the DF group were fed by surrogate mothers and not exposed to asphyxia or cold stress [18].

### Sample collection and tissue processing

After termination, all premature rats were euthanized by neck dislocation and laparotomies were performed. A 2.0-cm piece of distal ileum was removed and stained with hematoxylin and eosin (H&E) and used for RNA extraction. One-half of each intestinal specimen was fixed in 4.0% paraformaldehyde, paraffin embedded, cut into 4.0-μm sections, and stained with H&E to observe the histological changes under an optical microscope. The remaining one-half of each intestinal tissue was washed with cold normal saline (4.0 °C) and stored in liquid nitrogen for subsequent RNA analysis.

Histological changes in the ileum were evaluated by a blinded assessor using the following NEC scoring criteria [18]: 0 (normal), no damage; 1 (mild), slight submucosal and/or lamina propria separation; 2 (moderate), moderate separation of submucosa and/or lamina propria, and/or edema in submucosal and muscular layers; 3 (severe), severe separation of submucosa and/or lamina propria, and/or severe edema in submucosa and muscular layers, regional villi sloughing; 4 (necrosis), loss of villi and necrosis. Scores ≥2 represented NEC-like intestinal damage.

### RNA extraction and quantitative polymerase chain reaction

The mRNA expressions of TLR4, TOLLIP, and SIGIRR were evaluated using GAPDH as the endogenous gene control by real-time quantitative polymerase chain reaction (RT-qPCR). Total RNA was extracted from frozen ileal tissue using the HiPure Total RNA Mini Kit (Magen, Guangzhou, China) and following the manufacturer’s instructions. RNA integrity was verified by agarose gel electrophoresis. The purity and concentration of RNA were quantified using the Nanodrop ND-1000 UV Spectrophotometer (Thermo Fisher Scientific, Waltham, MA, USA).

Reference Sequence Database access codes were as follows: NM_019178 (TLR4), NM_001109668 (TOLLIP), NM_001024887 (SIGIRR), and NM_017008.4 (GAPDH). TLR4 upstream primers were 5′-TATCCAGAGCCGTTGGTGTATT-3′ and the downstream primers were 5′-AATGAAGATGATGCCAGAGCG-3′. The length of the PCR product was 85 base pairs (bp). TOLLIP upstream primers were 5′-CCAAGTGGAGGACGAGTGGTAT -3′ and the downstream primers were 5′-GCTTCAAGCACAGAACGGATT-3′. The length of the PCR product was 330 bp. SIGIRR upstream primers were 5′-TGGTGAACCTGAGTCGCTGTC -3′ and the downstream primers were 5′-CCCTCCACGGGTCTCTATTG-3′. The length of the PCR product was 310 bp. GAPDH upstream primers were 5′-TTCCTACCCCCAATGTATCCG-3′ and the downstream primers were 5′-CATGAGGTCCACCACCCTGTT-3′. The length of the PCR product was 281 bp. PCR primers were designed using the Primer-BLAST tool (NCBI, Bethesda, MD, USA) and synthesized by Goodbio Technology Company (Wuhan, China). cDNA was synthesized using the PrimeScript™ RT Reagent Kit (TaTaRa, Dalian, China) and following the manufacturer's instructions.

mRNA levels were detected in triplicate using SYBR Premix Ex Taq™ Kit (Takara, Dalian, China) on a Mx3000P (Stratagene, California, La Jolla, CA, USA) with a 20-μL RT-qPCR reaction mixture. The amplification reaction was started at 95 °C for 60 s for initial denaturation, followed by 40 consecutive PCR cycles at 95 °C for 5.0 s, and 60 °C for 30 s. The fold changes in specific gene expression were calculated using the ##CT method [22].

### Statistical analyses

The statistical analyses were conducted using SPSS13.0 software (Armonk, NY, USA). mRNA expression of TLR4, TOLLIP and SIGIRR are presented as the mean  ±  SE. The difference in expression among DF, FF, and FB groups was analyzed using one-way analysis of variance followed by the Student–Newman–Keul multiple comparisons test. The Mann–Whitney test was used to compare different pathological ileum scores between the FF and FB groups. Survival time was analyzed using Kaplan–Meier analysis (with the log-rank test). The incidence of NEC-like intestinal injury was evaluated using the Fisher's exact test. *P*  <  0.05 was considered statistically significant. All analyses were two-tailed tests.

## Results

### General status

**Fig. 1:**
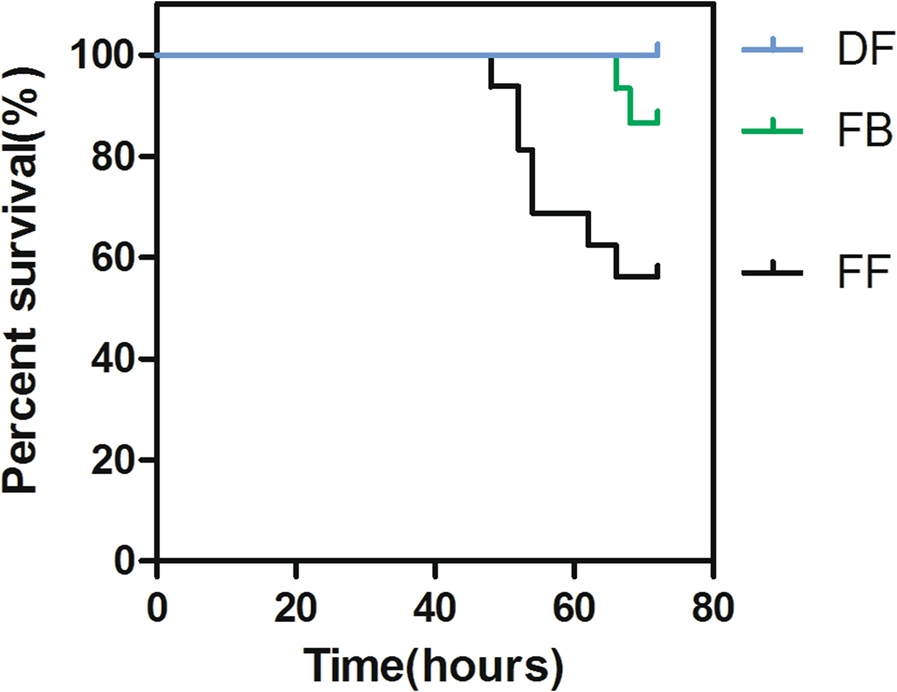
Kaplan–Meier survival analysis. The mean survival time was 64.8 h (64.8 ± 2.3) in the formula fed (FF) group and 71.3 h (71.3 ± 0.5) in the formula + *Bifidobacterium adolescentis* (FB) group. Rats in the FB group had a significantly better 72-h survival rate than in the FF group (*P*  <  0.05)

There were no statistically significant differences in birth weight among the three groups: Throughout the entire experiment, the premature rats in the DF group increased their weight. The rats in the FB group maintained their body weight but those in the FF group gradually lost body weight, one symptom of the disease. The mean body weight of rats in the DF, FF group and FB groups was 7.90  ±  0.50 g, 5.58  ±  0.73 g and 6.40  ±  0.67 g, respectively, with a statistically significant difference at the end of the 72 h (*P*  <  0.001). The other NEC-like symptoms appeared between 48 and 72 h; these were abdominal distension and diarrhea, which were similar to those described in a previous study [23]. The mean survival time was 64.8 h (64.8 ± 2.3) in the FF group and 71.3 h (71.3 ± 0.5) in the FB group. The 72-h survival rates in the FF, FB and DF groups were 56.3 (9/16), 86.7 (13/15) and 100% (14/14), respectively. Rats in the FB group had a significantly better survival rate than those in the FF group (*P* = 0.005, Fig. 1).

### Effects of *B. adolescentis* on the incidence and severity of NEC-like injury in neonatal rats

**Fig. 2:**
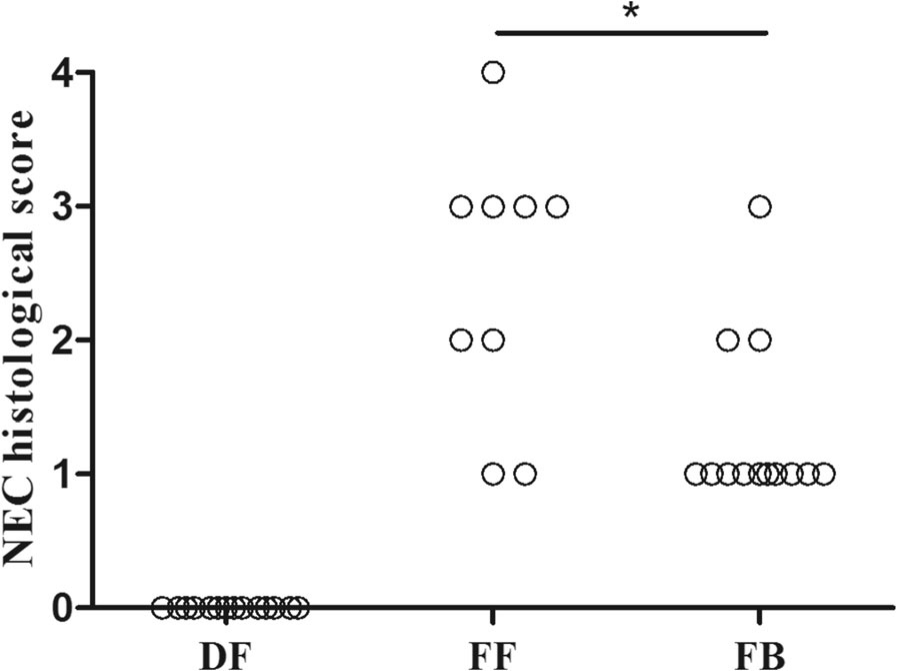
Necrotizing enterocolitis (NEC) histological score of ileum in each group. Histological scoring of the terminal ileum of premature newborn rats in dam-fed (DF), formula-fed (FF), and formula + *Bifidobacterium adolescentis* (FB) groups (DF, *n* = 14; FF, *n* = 9; FB, *n* = 13). NEC-like intestinal damage was defined as scores  ≥  2. *indicates *P*  <  0.05

**Fig. 3:**
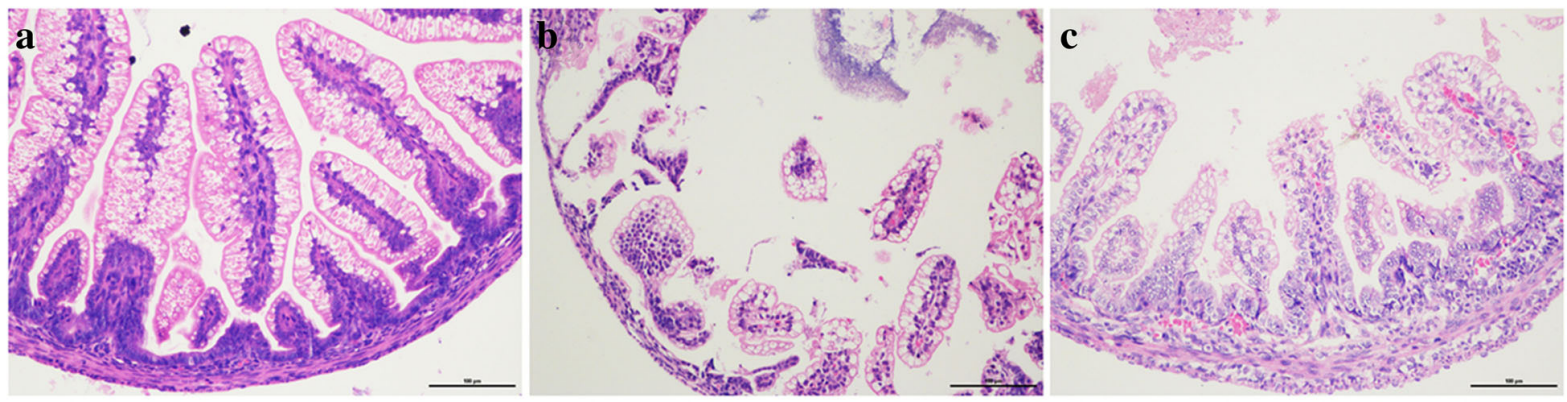
Histological changes of ileum in premature newborn rats. The representative images from each group are presented. (**a**) The structure of intestinal mucosa is intact in the dam-fed (DF) group; (**b**) the structure of ileum villus was broken and even lost in the formula-fed (FF) group; (**c**) the ileum of premature rats in formula + *Bifidobacterium adolescentis* (FB)group had less necrotic ilea and lost villi. Scale bar represents 100 μm. Original magnification: 200 ×

The ileal histological score was lower in the FB group (median, 1.0) than in the FF group (median, 3.0) (*P* = 0.013). Administration of *B. adolescentis* significantly decreased the rate of NEC-like intestinal injury from 77.8% (7/9) in the FF group to 23.1% (3/13) in the FB group (*P* = 0.027). In the DF group, the incidence of NEC-like intestinal injury was 0% (0/14) and the median histological score was 0 (Fig. 2). Representative images from each group are shown in Fig. 3a, b and c.

### Evaluation of mRNA expression of TLR4, TOLLIP, and SIGIRR in the ileum tissues

**Fig. 4:**
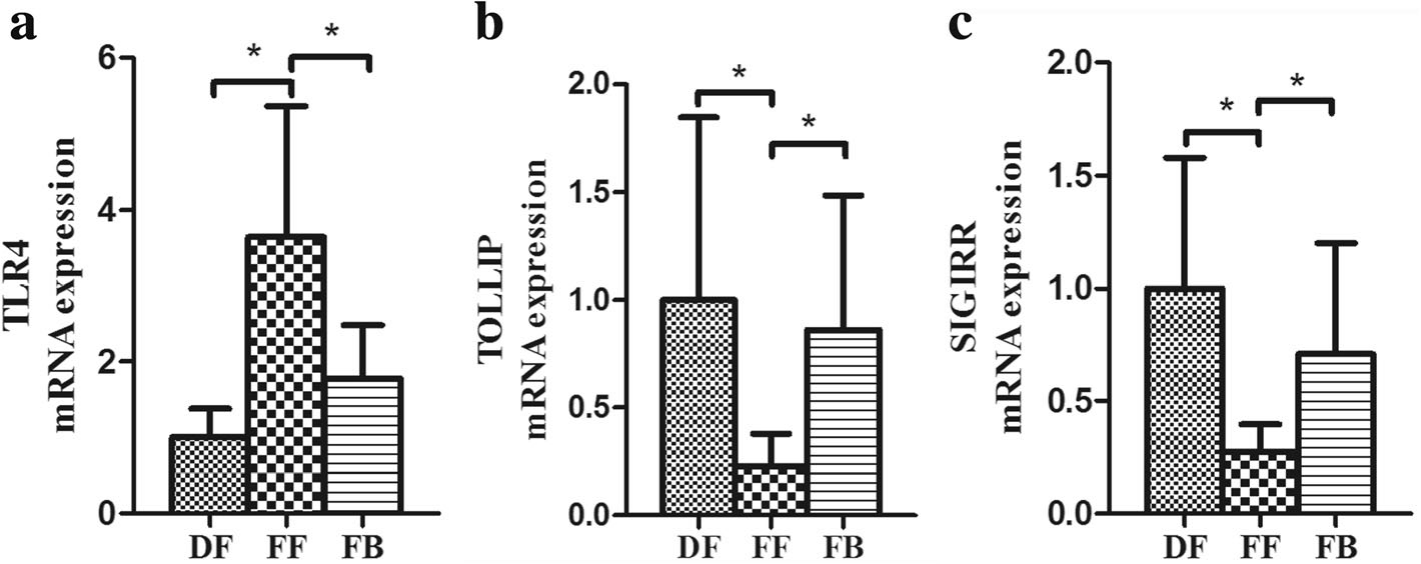
Fold changes in the relative mRNA expression of TLR4, TOLLIP, and SIGIRR of the ileum of premature neonatal rats in dam-fed (DF), formula-fed (FF), and formula + *Bifidobacterium adolescentis* (FB) groups. The mRNA expression of TLR4 (**a**), TOLLIP (**b**), and (**c**) SIGIRR(B) in DF, FF, and FB groups (DF, *n* = 14; FF, *n* = 9; FB, *n* = 13) is shown. The mRNA expression for the DF group is set a value of 1.0, and mRNA levels for the FF and FB groups are determined relative to this number. Values are presented as the mean  ±  SE. *indicates *P*  < 0.05

The ileum mRNA expression of TLR4 increased by 3.6 fold in the FF group compared with that in the DF group (*P* < 0.05), while the expression of TLR4 mRNA decreased by 2.0 fold as a result of *B. adolescentis* treatment (*P* < 0.05) (Fig. 4a). In contrast, the mRNA expression of TOLLIP and SIGIRR decreased 4.3 and 3.7 fold in the FF group, respectively compared with those in the DF group (*P* < 0.05) (Fig. 4b and c). Administration of *B. adolescentis* significantly increased mRNA expression of TOLLIP and SIGIRR by 3.7 and 2.6 fold, respectively (*P* < 0.05) (Fig. 4b and c). There was no statistical difference in mRNA expression of TLR4, TOLLIP, or SIGIRR between the FB and DF groups (*P* > 0.05).

## Discussion

The model used is a well-established NEC model [24] that is based on the induction of intestinal injury using formula feeding and hypoxia-hypothermia treatment as two critical instigating factors on premature rats [24]. Previous studies [23, 25] suggested that all dam-fed pups or pups without exposure to hypoxia-hypothermia showed normal intestinal histology or less intestinal injury. We recognize that NEC development in human infants is more complex, thus the pathophysiology of a rat model might be different from that seen in humans; however, we used this model to explore the protective effect of *B. adolescentis* on pathophysiologic mechanisms associated with NEC.

In this study, prophylactic *B. adolescentis* significantly alleviated ileum damage in the NEC rat model, and also inhibited the increase in TLR4 expression and the decrease in TOLLIP and SIGIRR expression in the intestine. These results indicated that *B. adolescentis* protects premature rats from intestinal injury from hypoxia and cold stress, the mechanism of which might be by reducing the expression of TLR4 and increasing the expression of TOLLIP and SIGIRR.

Although the pathogenesis of NEC is not entirely known [1],the theory that inappropriate microbial colonization in the intestine induces the immature intestine to mount an excessive inflammatory response that results in tissue injury and necrosis is a consideration [1]. IL-8 increases in response to inflammatory stimuli in immature enterocytes compared with mature enterocytes [26]. It was reported that the same effect is seen in animal models, intestinal explants, and xenografts [27]. Clinical observation has shown that patients with NEC, especially infants who need surgery, have much higher levels of proinflammatory cytokines [28].

TLR4 is activated by LPS, a Gram-negative bacteria in the cell wall and the main ligand for TLR4, then signal through MyD88 to promote the production and release of proinflammatory cytokines though NF-κB [7]. Myd88 is a TLR4 adaptor molecule that is recruited to TLR4 and subsequently recruits IRAK [7]. TOLLIP and SIGIRR impair signaling from TLR4 by suppressing IRAK, which results in reducing the synthesis of proinflammatory cytokines [14]. TOLLIP binds to IRAK and inhibits IRAK phosphorylation [16] to downregulate the TLR4 signal. SIGIRR impairs TLR4 signaling that leads to NF-κB activation by competing with MyD88 and IRAK recruitment at the Toll-IL-1-receptor (TIR) domain [29], which participates in the interaction of SIGIRR with TLR4 [30]. Nanthakumar [8] suggested that the inappropriate inflammatory response in the immature intestine is a result of the imbalance between TLR4 and its negative regulators. TLR4 and chemokine expression is greater in both fetal and NEC enterocytes than that in mature human enterocytes [8]. SIGIRR and TOLLIP expression, which inhibits the inflammatory reaction, is lesser in both immature and NEC enterocytes than that in mature enterocyte [8]. In this study, we demonstrated that the level of TLR4 increases while the level of negative effector molecules decreases. Accordingly, it is reasonable to speculate that NEC is related to TLR4 and its negative regulators.

Many treatments have been proposed to prevent NEC but few have proved promising [1]. The most recently reported multicenter trial suggested that prophylactic probiotic treatment decreases the incidence of NEC [19, 31, 32]. *Bifidobacterium* is one type of Gram-positive probiotic that quantitatively colonizes in the intestine more than Gram-negative pathogenic bacteria [19, 31, 32]. Bergmann et al. [33] reported that the administration of *B. infantis* inhibits an increase in intestinal permeability and reduces NEC incidence in the NEC neonatal mouse model. B. *longum subsp. infantis* decreases the level of proinflammatory cytokines, which are important in NEC pathogenesis [34]. It was also reported that probiotics attenuates TLR4 expression in the NEC rat model at the protein level [34]. Recently, Ganguli et al. [4] reported that TOLLIP and SIGIRR mRNA levels increase and TLR4 mRNA level decreasese when immature human intestinal xenografts incubate with conditioned media of *B. infantis*, and that TOLLIP and SIGIRR mRNA expressions also increase in NEC intestinal epithelial cells. In this study, we demonstrated that *B. adolescentis* confers significant protection against intestinal injury in the NEC premature rat model and alter the expression of TLR4, TOLLIP and SIGIRR, all factors in the NF-κB signaling pathway; therefore, we speculate that *B. adolescentis* modulates the NF-κB signaling pathway by upregulateing TOLLIP and SIGIRR, and decreasing TLR4 to protect against intestinal injury.

Probiotics exert their anti-inflammatory effects to reduce the incidence of NEC, but the mechanism is not known. These effects might be mediated by products secreted by probiotic bacteria. Probiotic conditioned media contain secreted products, free of intact bacteria, and it was recently reported that this media reduces NEC-like intestinal damage in an animal model [35]. Probiotic conditioned media inhibites the NF-κB signaling pathway and its proinflammatory cytokine production [36], which contributes to attenuating the intestinal inflammatory response [36]. It was also reported that *Bifidobacterium-*conditioned media reduces the level of TLR2 and TLR4 in xenograft epithelium and increases the level of SIGIRR and TOLLIP [4]; therefore, it is reasonable to speculate that *Bifidobacterium* species might alleviate intestinal injury through their secreted products, although what the probiotic conditioned media contains is not clear. Ganguli [4] suggested that a protein or nucleic acid from 5.0 to 10 kDa, might be in the media; however, Shiou [35] suggested that it is not a protein. This controversy will require further experiments, which is beyond the scope of the current study.

Another possible bioactive effector that protects premature rats from NEC might be some component of killed bacteria [37]. It was recently reported that heat-killed probiotics are as effective as live probiotics in maintaining the intestinal barrier [38] and that they can alleviate intestinal inflammation [39]. Both TOLLIP and SIGIRR are closely related to the intestinal barrier. Intestinal permeability in TOLLIP-deficient [16] and SIGIRR-deficient mice [40] treated with DSS increases compared with that in wild-type controls. It was also reported that the heat-killed probiotics alleviate intestinal inflammation through the downstream TLR4 pathway [41]. Dead *Lactobacillus*, which were isolated from newborn infant feces and breast milk, as well as live bacteria, activate TLR4-associated negative regulators, including TOLLIP, SOCS1, SOCS3 and IκBα [41]. It appears that the protective effect of *B. adolescentis* on NEC might be the result of upregulating SIGIRR and TOLLIP using some component of dead *B. adolescentis*.

## Conclusions

We believe that prophylactic *B. adolescentis* protects against intestinal injury in a well-established premature newborn rat model, and inhibits the increase in intestinal TLR4 and the decrease in TOLLIP and SIGIRR expression. These results help to gather insight into the mechanisms behind the protective effects of probiotics in the intestine; however, in this study the increases in the expression of both SIGIRR and TOLLIP were demonstrated only in the NEC rat model, further studies are required to better determine the mechanisms underlying the beneficial effects of probiotics.

## Abbreviations

DF: Dam fed group
DSS: Dextran sodium sulfate
FB: Formula + *Bifidobacterium adolescentis* group
FF: Formula Fed group
GAPDH: Glyceraldehyde-3-phosphate dehydrogenase
IL: Interleukin
IRAK: IL-1 receptor-associated kinase
NEC: Necrotizing enterocolitis
qPCR: Quantitative polymerase chain reaction
RNA: Ribonucleic acid
SD: Sprague-dawley
SIGIRR: Single Ig IL-1-Related Receptor
TIR: Toll-IL-1-receptor
TLR: Toll-like receptor
TOLLIP: Toll Interacting Protein
VLBW: Very low birth weight
